# A mouse model of Weaver syndrome displays overgrowth and excess osteogenesis reversible with KDM6A/6B inhibition

**DOI:** 10.1172/jci.insight.173392

**Published:** 2024-01-09

**Authors:** Christine W. Gao, WanYing Lin, Ryan C. Riddle, Priyanka Kushwaha, Leandros Boukas, Hans T. Björnsson, Kasper D. Hansen, Jill A. Fahrner

**Affiliations:** 1 Department of Genetic Medicine,; 2 Department of Molecular Biology and Genetics, and; 3 Department of Orthopaedic Surgery, Johns Hopkins University School of Medicine, Baltimore, Maryland, USA.; 4 Department of Orthopaedics, University of Maryland School of Medicine, Baltimore, Maryland, USA.; 5 Research and Development Service, Baltimore Veterans Administration Medical Center, Baltimore, Maryland, USA.; 6 Department of Biostatistics, Johns Hopkins University School of Public Health, Baltimore, Maryland, USA.; 7 Department of Pediatrics, Johns Hopkins University School of Medicine, Baltimore, Maryland, USA.; 8 Faculty of Medicine, University of Iceland, Reykjavík, Iceland.; 9 Landspítali University Hospital, Reykjavík, Iceland.; 10 Department of Biomedical Engineering, Johns Hopkins University School of Medicine, Baltimore, Maryland, USA.

**Keywords:** Genetics, Epigenetics, Genetic diseases

## Abstract

Weaver syndrome is a Mendelian disorder of the epigenetic machinery (MDEM) caused by germline pathogenic variants in *EZH2*, which encodes the predominant H3K27 methyltransferase and key enzymatic component of Polycomb repressive complex 2 (PRC2). Weaver syndrome is characterized by striking overgrowth and advanced bone age, intellectual disability, and distinctive facies. We generated a mouse model for the most common Weaver syndrome missense variant, EZH2 p.R684C. *Ezh2^R684C/R684C^* mouse embryonic fibroblasts (MEFs) showed global depletion of H3K27me3. *Ezh2^R684C/+^* mice had abnormal bone parameters, indicative of skeletal overgrowth, and *Ezh2^R684C/+^* osteoblasts showed increased osteogenic activity. RNA-Seq comparing osteoblasts differentiated from *Ezh2^R684C/+^*, and *Ezh2^+/+^* BM-mesenchymal stem cells (BM-MSCs) indicated collective dysregulation of the BMP pathway and osteoblast differentiation. Inhibition of the opposing H3K27 demethylases KDM6A and KDM6B substantially reversed the excessive osteogenesis in *Ezh2^R684C/+^* cells both at the transcriptional and phenotypic levels. This supports both the ideas that writers and erasers of histone marks exist in a fine balance to maintain epigenome state and that epigenetic modulating agents have therapeutic potential for the treatment of MDEMs.

## Abstract 2

An EZH2 missense mouse model of Weaver syndrome exhibits skeletal overgrowth, excess osteogenesis, and a characteristic transcriptomic profile that is reversed with a KDM6A/6B inhibitor.

## Introduction

Mendelian disorders of the epigenetic machinery (MDEMs) result from germline pathogenic variants in writers, erasers, and readers of epigenetic marks as well as chromatin remodelers (1, 2). The characteristic feature of this class is a combination of growth abnormality (either overgrowth or undergrowth; seen in 74% of these disorders) and developmental delay/intellectual disability (present in 85% of disorders) (3). To date, at least 85 MDEMs have been described, yet treatment still only consists of symptomatic management and preventative screening for known complications (3). Despite being monogenic disorders, MDEMs are thought to cause multisystemic findings through widespread epigenomic dysregulation and consequent transcriptomic perturbation. This has been supported by ATAC-Seq and RNA-Seq studies in both animal and cell line models of several MDEMs, showing alterations to chromatin accessibility and gene expression (4–6). Recently, peripheral blood DNA methylation signatures have also been identified for many MDEMs and are now not only approved for diagnosis, but they also can reliably differentiate pathogenic variants from benign (7–11). This reinforces the idea that the core etiology of MDEMs lies in their effects upon the epigenome, which is a unifying quality of MDEMs.

Weaver syndrome (MIM 277590) is a MDEM with cardinal signs of overgrowth, developmental delay/intellectual disability, and characteristic facial appearance as well as advanced osseous maturation (12–16). With the advent of whole-exome sequencing, Tatton-Brown et al. and Gibson et al. traced the molecular etiology of Weaver syndrome to heterozygous pathogenic variants in *EZH2*, which encodes the primary H3K27 methyltransferase and core component of the Polycomb repressive complex 2 (PRC2) (14, 15). PRC2 is highly conserved across plants, fungi, and animals and is involved in fundamental processes such as cell differentiation, development, and cell cycle control (17). Pathogenic variants in other components of PRC2 partially phenocopy Weaver syndrome: Cohen-Gibson syndrome (*EED* variants; MIM 617561) (18–20) and Imagawa-Matsumoto syndrome (*SUZ12* variants; MIM 618786) (21–23) also have overgrowth, advanced bone age, and developmental delay/intellectual disability as central features. Interestingly, all 3 PRC2 MDEMs share a peripheral blood DNA methylation signature (10), suggesting a common mechanism of disease linked to PRC2 dysfunction.

The exact mechanism by which PRC2 dysfunction translates to an overgrowth phenotype at the organismal level is still being explored. Long bone growth is driven by endochondral ossification at cartilaginous growth plates (24). Chondrocytes at the growth plate undergo rapid proliferation and hypertrophy, during which the cells secrete an abundance of extracellular matrix. This matrix acts as a scaffold for invasion by vasculature, osteoclasts, and osteoblasts. While osteoclasts resorb old matrix, osteoblasts lay down new bone. The fine orchestration of these cell types continues to shape and remodel the bone structure throughout life and is affected by changes in lineage commitment, proliferation, and development. This process is also regulated by a plethora of endocrine signals, such as growth hormone (GH) and thyroid hormone, as well as local factors such as Indian hedgehog (IHH), bone morphogenetic proteins (BMPs), and insulin-like growth factors (IGFs), all which in turn influence intracellular transcription regulatory networks. *EZH2* is downregulated within 3 days after the onset of osteogenic differentiation in mesenchymal stem cells (MSCs) (25). Conditional KO of *Ezh2* in the MSC lineage leads to severe skeletal patterning defects and overexpression of cyclin-dependent kinase (CDK) inhibitors, but siRNA knockdown or pharmacological inhibition of *Ezh2* in MSCs also increases osteogenic marker expression (25, 26). This suggests that *Ezh2* is necessary for early proliferation of osteogenic precursors yet simultaneously suppresses osteoblast maturation. However, these conditional KO and complete inhibition models do not capture the growth patterns of Weaver syndrome, which generally originates from constitutional heterozygous pathogenic missense variants.

To investigate the mechanisms behind skeletal overgrowth in Weaver syndrome, we generated a constitutional missense mouse model, *Ezh2^R684C/+^*. This heterozygous model both allowed in vivo skeletal profiling and provided a source of primary cells for in vitro studies of osteoblast differentiation. Here, using μ-CT, we describe a skeletal overgrowth phenotype in *Ezh2^R684C/+^* mice that is reminiscent of Weaver syndrome. Both our in vivo labeling and in vitro differentiation assays suggest that excessive osteogenesis by the osteoblast lineage bears responsibility for the overgrowth, and we identify a distinct transcriptional profile in *Ezh2^R684C/+^* osteoblasts. Finally, we found reversal of both the osteogenic phenotype and transcriptional perturbations of *Ezh2^R684C/+^* osteoblasts following treatment with an epigenetic modulator, GSK-J4. These findings contribute to the growing body of literature indicating that MDEMs could one day be treated by addressing their epigenetic etiology.

## Results

### A recurrent Weaver syndrome missense variant causes loss of H3K27me3 methyltransferase activity.

**Figure 1:**
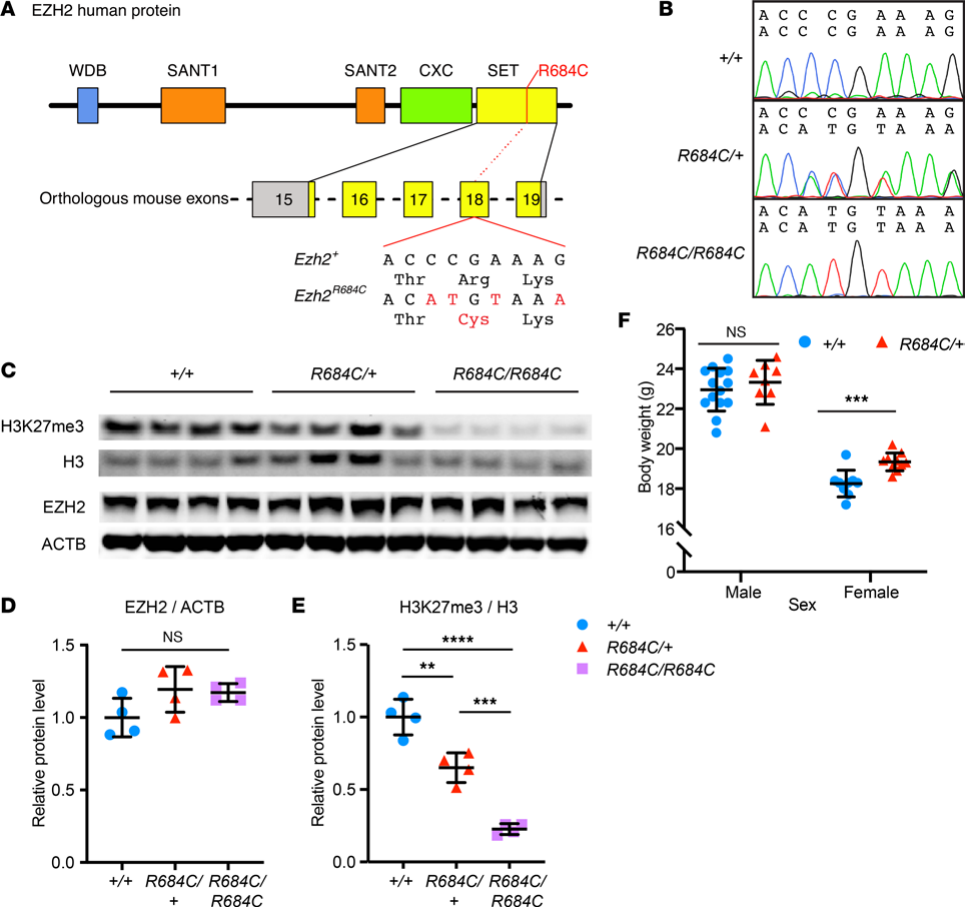
EZH2 R684C leads to reduced H3K27me3 catalysis and overgrowth in female mice. (**A**) Four base changes were introduced into exon 18 of *M*. *musculus* *Ezh2*, changing codon CGA (Arg 679) in the catalytic SET domain (yellow) to TGT (Cys) and introducing 2 silent mutations to create an Nsp1 restriction site for genotyping. At the protein level, this corresponds to *H*. *sapiens* EZH2 p.R684C. (**B**) Chromatogram traces for E14.5 mouse embryonic fibroblasts (MEFs) that are WT at the *Ezh2* locus (*+/+*), heterozygous (*R684C/+*), or homozygous for the *R684C* variant allele (*R684C/R684C*). (**C**) Western blot detecting EZH2 and ACTB in whole-cell lysates from *Ezh2^+/+^*, *Ezh2^R684C/+^*, and *Ezh2^R684C/R684C^* MEFs, as well as H3K27me3 and H3 in corresponding histone-extracted samples. H3 and ACTB served as loading controls. (**D**) Quantification of the Western blot shows that EZH2 protein levels do not differ between genotypes, after normalization to ACTB loading control. One-way ANOVA. (**E**) Relative to *Ezh2^+/+^*, the ratio of H3K27me3 to H3 is reduced to a mean of 0.65 in *Ezh2^R684C/+^* and 0.23 in *Ezh2^R684C/R684C^*. **P < 0.01, ***P < 0.001, ****P < 0.0001, 1-way ANOVA with Tukey’s multiple-comparison test. For **D** and **E**, blue circles represent *Ezh2^+/+^*; red triangles represent *Ezh2^R684C/+^*; purple squares represent *Ezh2^R684C/R684C^*. *n* = 4 in each group. (**F**) Female *Ezh2^R684C/+^* mice have increased body weight at 8 weeks of age compared with female *Ezh2^+/+^* littermates. *Ezh2^+/+^* males, *n* = 14; *Ezh2^+/+^* females, *n* = 9. *Ezh2^R684C/+^* males, *n* = 8; *Ezh2^R684C/+^* females, *n* = 10. Blue circles represent *Ezh2^+/+^*; red triangles represent *Ezh2^R684C/+^*. ***P < 0.001, unpaired Student’s *t* test. Data represent mean ± 1 SD.

*EZH2* c.2050 C>T (p.R684C) is the most common heterozygous pathogenic variant in unrelated patients with Weaver syndrome (14, 16) and lies within the catalytic Su(var)3-9, Enhancer of Zeste, and Trithorax (SET) methyltransferase domain, which is highly conserved across species (27–30). Using CRISPR-Cas9 gene editing, we generated a mouse model bearing the orthologous *Ezh2* missense variant (Figure 1, A and B). We refer to the edited allele as *Ezh2^R684C^* for simplicity. *Ezh2^R684C/+^* pups were born full-term at Mendelian ratios (Supplemental Table 1, A–C; supplemental material available online with this article; https://doi.org/10.1172/jci.insight.173392DS1). This suggests that a severe disruption to global H3K27me3 is unlikely in the heterozygous state, since H3K27me3 is a critical mark for transcriptional regulation, maintenance of facultative heterochromatin, and thus initiation and preservation of cell differentiation state (31). Embryos homozygous for the R684C variant (*Ezh2^R684C/R684C^*) were viable through E14.5 but ultimately did not survive to birth (Supplemental Table 1, A and B).

*Ezh2^R684C/R684C^* mouse embryonic fibroblasts (MEFs) isolated at E14.5 had WT levels of EZH2 protein expression (Figure 1, C and D). This indicated that the R684C allele produces a full-length protein product, albeit catalytically compromised, as corresponding H3K27me3 levels were drastically decreased (Figure 1, C and E). A minimal amount of residual H3K27me3 in *Ezh2^R684C/R684C^* MEFs may be due to EZH1, a homolog of EZH2 that has low levels of H3K27me3 methyltransferase activity (32). The decrease in global H3K27me3 levels for *Ezh2^R684C/+^* MEFs was approximately half the respective decrease for *Ezh2^R684C/R684C^* MEFs when both are normalized to *Ezh2^+/+^* MEFs (Figure 1, C and E), suggesting halved gene dosage in *Ezh2^R684C/+^* cells. Our data indicate that R684C is a loss-of-function variant, or possibly a severe hypomorph, that interferes with SET domain catalysis but does not affect protein stability. Although this lowered global H3K27 methyltransferase activity does not reduce the viability of *Ezh2^R684C/+^* pups, as noted above, altered H3K27me3 levels at specific genomic loci likely contribute to the phenotype of *Ezh2^R684C/+^* mice through dysregulation of gene expression.

### Ezh2^R684C/+^ mice exhibit a skeletal overgrowth phenotype.

**Figure 2:**
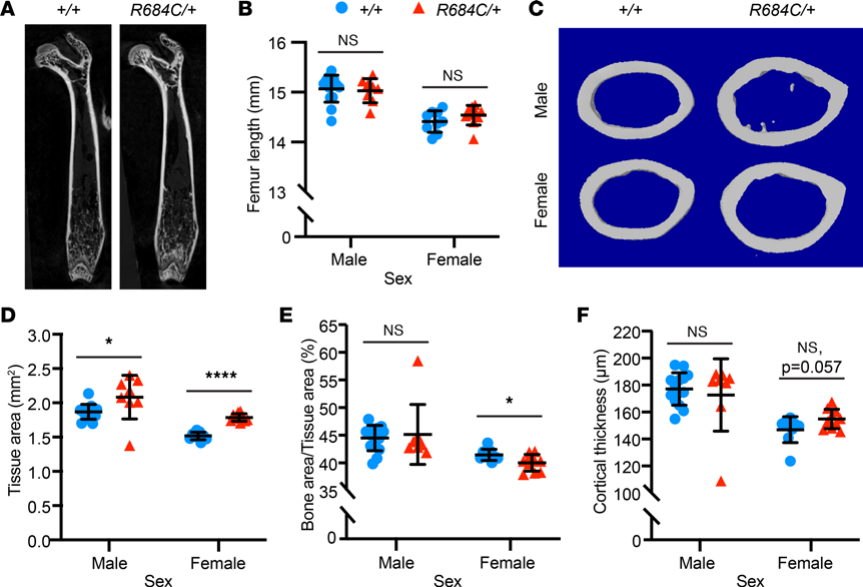
*Ezh2^R684C/+^* mice have altered cortical bone parameters. (**A**) μ-CT of *Ezh2^+/+^* and *Ezh2^R684C/+^* femurs in the coronal plane. (**B**) Femur lengths do not differ between *Ezh2^+/+^* and *Ezh2^R684C/+^* mice for either sex. (**C**) μ-CT reconstructions of cortical bone regions of interest at the femoral middiaphysis. (**D**) Tissue area is notably increased in *Ezh2^R684C/+^* mice of both sexes. (**E**) *Ezh2^R684C/+^* female mice have a lower bone area/tissue area percentage. (**F**) Female *Ezh2^R684C/+^* mice have a trend toward higher cortical thickness (n.s., *P* = 0.057). *Ezh2^+/+^* males, *n* = 14; *Ezh2^+/+^* females, *n* = 9. *Ezh2^R684C/+^* males, *n* = 8; *Ezh2^R684C/+^* females, *n* = 10. Blue circles represent *Ezh2^+/+^*; red triangles represent *Ezh2^R684C/+^*. *P < 0.05, ****P < 0.0001, unpaired Student’s *t* test. Data represent mean ± 1 SD.

Because overgrowth is the most striking feature of Weaver syndrome, we first focused on this aspect of the phenotype. Similar to individuals with Weaver syndrome, *Ezh2^R684C/+^* mice displayed overgrowth. Female *Ezh2^R684C/+^* mice showed higher body weight compared with *Ezh2^+/+^* littermates at 8 weeks of age (*P* = 0.0006) (Figure 1F). This subtle overgrowth trend was observed through 22 weeks in female mice, although we did not see similar growth differences in male *Ezh2^R684C/+^* mice (Figure 1F and Supplemental Figure 1). Direct measurements and high-resolution μ-CT imaging did not reveal a difference in femur and tibia length between 8-week-old *Ezh2^R684C/+^* and *Ezh2^+/+^* mice (Figure 2, A and B, and Supplemental Figure 2A), nor did we observe differences in total body length (Supplemental Figure 2B) or trabecular bone structure in the distal femur (Supplemental Figure 2, C–E). However, *Ezh2^R684C/+^* mice did exhibit striking alterations in cortical bone structure at the femoral middiaphysis. Cross-sectional tissue area was notably increased in both male and female *Ezh2^R684C/+^* mice when compared with *Ezh2^+/+^* littermates (*P* = 0.032 and *P* < 1 × 10^–6^, respectively) (Figure 2, C and D), and this increase led to a decrease in the bone area/tissue area percentage in females (*P* = 0.028) (Figure 2E). Female *Ezh2^R684C/+^* mice also had a trend toward increased cortical thickness (*P* = 0.057) (Figure 2F).

**Figure 3:**
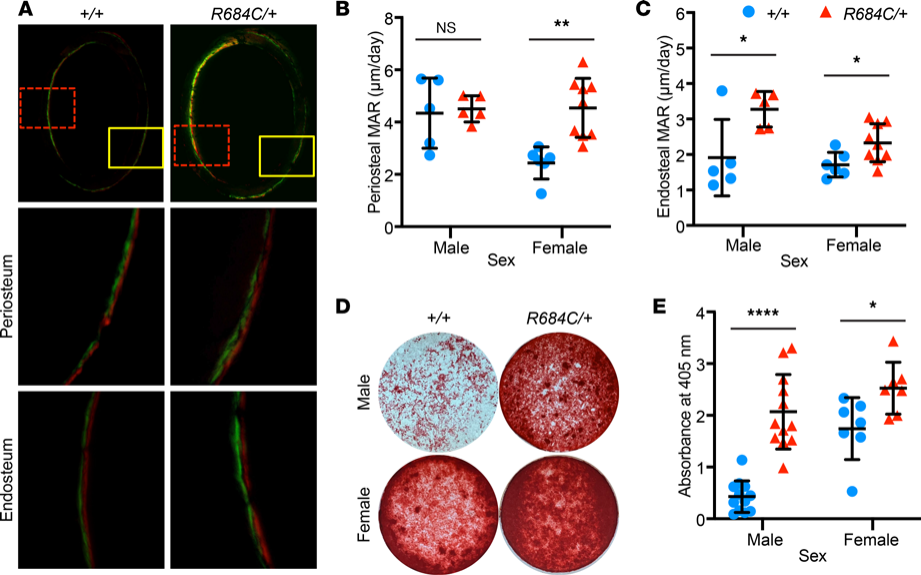
Osteoblast activity is increased in *Ezh2^R684C/+^* mice. (**A**) Representative images of double-fluorescence in vivo labeling at the femoral middiaphysis, in the transverse plane (4× magnification, top panels). Green, calcein; red, Alizarin red. Solid yellow boxes mark the regions for periosteal measurements (20× magnification, center panels); dashed red boxes mark the regions for endosteal measurements (20× magnification, bottom panels). (**B** and **C**) Mineral apposition rate (MAR) is increased at the periosteum in females only (**B**) and at the endosteum for both sexes (**C**). *Ezh2^+/+^* males, *n* = 5; *Ezh2^+/+^* females, *n* = 6. *Ezh2^R684C/+^* males, *n* = 5; *Ezh2^R684C/+^* females, *n* = 9. (**D**) Alizarin red staining of osteoblasts following 21 days of in vitro differentiation from primary murine BM-MSCs isolated from *Ezh2^R684C/+^* and *Ezh2^+/+^* mice. Representative whole-well images taken from a 24-well plate. (**E**) *Ezh2^R684C/+^* cells of either sex have higher uptake of Alizarin red, as quantified by absorbance at 405 nm. *Ezh2^+/+^* males, *n* = 12; *Ezh2^+/+^* females, *n* = 7; *Ezh2^R684C/+^* males, *n* = 12; *Ezh2^R684C/+^* females, *n* = 7. Blue circles represent *Ezh2^+/+^*; red triangles represent *Ezh2^R684C/+^*. *P < 0.05, **P < 0.01, ****P < 0.0001, unpaired Student’s *t* test. Data represent mean ± 1 SD.

To understand the basis for the cortical bone phenotype, we quantified the rate of bone formation by dynamic histomorphometry after sequential injection of calcein and Alizarin red at 5 weeks of age, when bone growth is rapid but prior to discernible overgrowth. The mineral apposition rate (MAR) is a direct measure of osteoblast activity. Compared with *Ezh2^+/+^* littermates, periosteal (Ps) MAR was markedly increased in female *Ezh2^R684C/+^* mice (*P* = 0.001) (Figure 3, A and B), and endosteal (Es) MAR was increased in both male and female *Ezh2^R684C/+^* mice (*P* = 0.033 and *P* = 0.028, respectively) (Figure 3, A and C). These findings indicate that the *Ezh2^R684C/+^* mouse model recapitulates certain overgrowth aspects of Weaver syndrome, particularly in female mice, and further suggest that cortical bone modeling is altered in *Ezh2^R684C/+^* mice, at least in part via excessive osteoblast activity.

### Ezh2^R684C/+^ bone marrow MSCs exhibit higher osteogenic potential in vitro.

To further investigate the pathogenic role of *Ezh2^R684C/+^* osteoblasts in vitro, we isolated murine BM-MSCs from 8- to 10-week-old mice and differentiated the cultures to osteoblasts (33). After 21 days of differentiation in identical conditions, *Ezh2^R684C/+^* cultures had substantially increased uptake of Alizarin red compared with *Ezh2^+/+^* in both males and females, indicative of enhanced deposition of calcium and mineralized bone matrix (males, *P* < 1 × 10^–6^; females, *P* = 0.021) (Figure 3, D and E). This occurred in the setting of stable cell numbers (data not shown). Quantitative PCR (qPCR) confirmed increased expression of osteogenic markers in *Ezh2^R684C/+^* osteoblasts from both sexes and even in the undifferentiated BM-MSC state for certain genes (Supplemental Figure 3, A–H), suggesting that *Ezh2^R684C/+^* BM-MSCs may be primed toward osteogenic differentiation. Altogether, these results support our in vivo findings and indicate that osteoblasts are critical to the *Ezh2^R684C/+^* phenotype, and they also demonstrate the use of BM-MSCs as an in vitro model of osteoblast differentiation for further studies.

### Osteoblasts differentiated from Ezh2^R684C/+^ BM-MSCs have a distinct gene expression profile.

**Figure 4:**
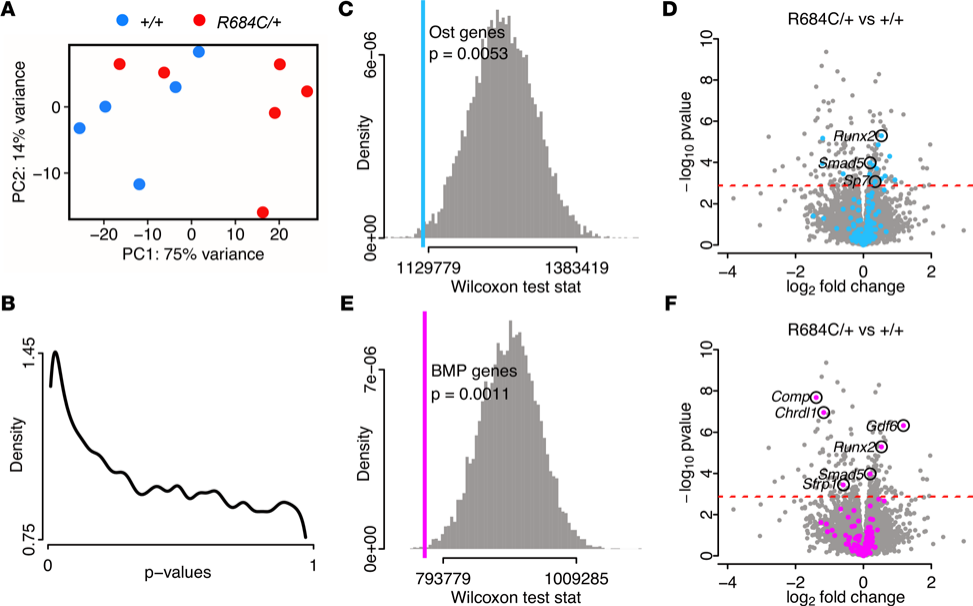
*Ezh2^R684C/+^* osteoblasts demonstrate transcriptional dysregulation of key osteogenic pathways. (**A**) Principal component analysis of *Ezh2^+/+^* (blue circles, *n* = 5) and *Ezh2^R684C/+^* (red circles, *n* = 6) RNA-Seq samples at day 14 of osteoblast differentiation. (**B**) Density plot of *P* values from differential expression analysis comparing *Ezh2^R684C/+^* versus *Ezh2^+/+^* samples, indicating an enrichment of low *P* values. (**C**) Wilcoxon test statistic for Mouse Genome Informatics (MGI) osteoblast differentiation genes (blue line, *P* = 0.0053) plotted over the simulated test statistic distribution for 10,000 random groupings of genes (gray). (**D**) Volcano plot for *Ezh2^R684C/+^* versus *Ezh2^+/+^* samples, with MGI osteoblast differentiation genes highlighted in blue. FDR = 0.1 (red dashed line). (**E**) Wilcoxon test statistic for MGI BMP pathway genes (magenta line, *P* = 0.0011) and simulated test statistic distribution for 10,000 random groupings of genes (gray). (**F**) Volcano plot comparing *Ezh2^R684C/+^* versus *Ezh2^+/+^* samples. MGI BMP pathway genes highlighted in magenta. FDR = 0.1 (red dashed line). Ost., osteoblast.

To identify gene expression changes responsible for enhanced osteogenesis in Weaver syndrome, we differentiated *Ezh2^R684C/+^* and *Ezh2^+/+^* BM-MSCs toward osteoblasts for 14 days and then performed transcriptome profiling with RNA-Seq. We chose this time point because transcriptional changes are usually evident prior to the appearance of phenotypic differences such as enhanced osteogenesis. Six female biological replicates were sequenced per genotype; principal component analysis indicated that samples clustered approximately according to genotype (Figure 4A). A histogram of gene-wise *P* values displayed a nonuniform distribution, with an overrepresentation of low *P* values (Figure 4B). Since a lack of differentially expressed genes (DEGs) would have resulted in a uniform distribution, this indicated the presence of differential gene expression in *Ezh2^R684C/+^* osteoblasts. In total, 194 genes were differentially expressed at the 10% FDR level (Supplemental Appendix 1), composed of 94 upregulated genes and 100 downregulated genes, referenced against *Ezh2^+/+^* osteoblasts (Supplemental Figure 4A). Of these, 36 genes had an absolute fold-change greater than 2, indicating a major difference in expression between *Ezh2^R684C/+^* and *Ezh2^+/+^*. We did not see a systemic upregulation of gene expression in *Ezh2^R684C/+^* cells as a result of the global reduction in H3K27me3. We verified that the DEGs we found in mouse osteoblasts roughly correspond to known human EZH2 target genes (Supplemental Figure 4B). Some incongruence is present, as expected, due to cell type and species differences. We also cross-validated our data with a publicly available RNA-Seq data set that investigated loss of EZH2 function through pharmacological inhibition with GSK-126 (34). This showed that genes affected by EZH2 inhibition are enriched among the population of genes perturbed in *Ezh2^R684C/+^* cells and vice versa (Supplemental Figure 4, C and D).

Because osteoblast differentiation is proximal to the mineralization phenotype observed upon in vitro Alizarin red staining as well as our prior in vivo dynamic histomorphometry data, we focused on this cell type. We utilized Gene Ontology (GO) annotations maintained by Mouse Genome Informatics (MGI) to compile a list of 201 genes involved in osteoblast differentiation (Supplemental Appendix 2), of which 179 genes were expressed in our data set. The *P* value ranks of these selected genes were significantly shifted from the *P* value rank of 179 genes randomly selected from our data set (*P* = 0.0053) (Figure 4C). This suggests that differentiation was collectively dysregulated in *Ezh2^R684C/+^* osteoblasts, even though not every individual gene had detectable dysregulation. Several key proosteogenic transcription factors were upregulated, such as *Runx2*, *Smad5*, and *Sp7*, and these genes warrant further study (Figure 4D). When we correlated the counts per million (CPM) for each of the osteoblast differentiation genes with the first principal component of the gene expression matrix (PC1), we found high correlation coefficients for this subset of genes compared with selecting random groups of genes (Supplemental Figure 5). This suggests that genes involved with osteoblast differentiation are a key contributor toward the variation captured by PC1 and, therefore, the distinction between *Ezh2^R684C/+^* and *Ezh2^+/+^* cells.

The BMP pathway is known to play an important role in osteogenesis, and we noticed an apparent abundance of associated genes among RNA-Seq hits. Therefore, we performed a similar analysis using an MGI-curated list of 161 BMP pathway genes (Supplemental Appendix 3) and discovered that this process is likewise dysregulated (*P* = 0.0011) (Figure 4E). *Gdf6*, *Runx2*, *Smad5*, *Comp*, *Chrdl1*, and *Sfrp1* are of particular interest for future investigation, as they are differentially expressed (Figure 4F). Notably, 38 genes are annotated as part of both the BMP pathway and osteoblast differentiation, as the processes are intricately intertwined. These pathway analyses provide further support, at a transcriptional level, of perturbed osteogenesis in *Ezh2^R684C/+^* osteoblasts. Overall, our RNA-Seq results also paint a profile of numerous shifts in transcription, acting in concert to produce a phenotype.

### Inhibition of H3K27me3 demethylases with GSK-J4 corrects excessive osteogenesis in vitro.

**Figure 5:**
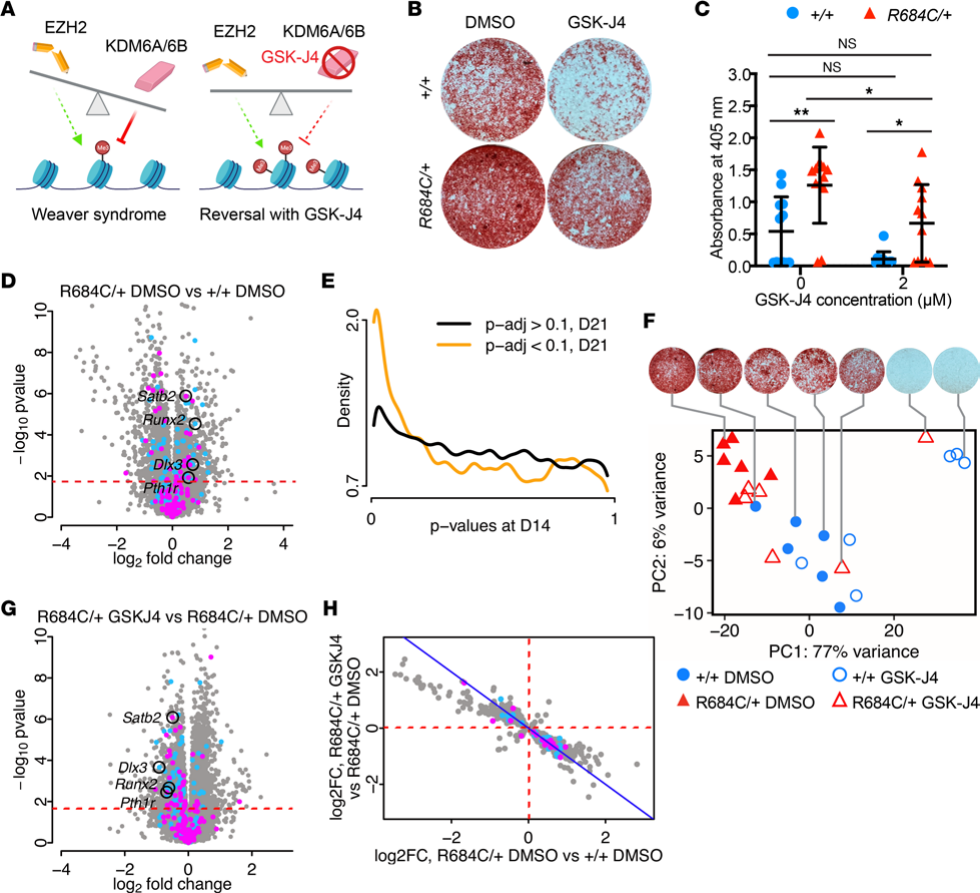
The KDM6A/6B inhibitor GSK-J4 substantially reverses the *Ezh2^R684C/+^* osteogenic phenotype and transcriptomic profile. (**A**) Balance hypothesis (1). Left: loss of EZH2 in Weaver syndrome allows for unopposed demethylase activity by KDM6A/6B. Right: inhibition of KDM6A/6B by GSK-J4 restores balance to the chromatin state. (**B**) Alizarin red staining of female *Ezh2^R684C/+^* and *Ezh2^+/+^* osteoblasts treated with 2 μM GSK-J4 or vehicle (DMSO). Cells were differentiated for 21 days from BM-MSCs. Representative whole-well images shown. (**C**) GSK-J4 treatment decreases Alizarin red staining in *Ezh2^R684C/+^* osteoblasts, as quantified by absorbance at 405 nm. *Ezh2^R684C/+^* osteoblasts continue to have higher absorbance than *Ezh2^+/+^*. No significant difference between *Ezh2^+/+^* vehicle-treated and *Ezh2^R684C/+^* GSK-J4–treated osteoblasts. Blue circles: *Ezh2^+/+^* females, *n* = 12. Red triangles: *Ezh2^R684C/+^* females, *n* = 12. *P < 0.05, **P < 0.01, 2-way ANOVA with Tukey’s multiple-comparison test. Data represent mean ± 1 SD. (**D**) Volcano plot of day 21 RNA-Seq, displaying log_2_ fold changes in the *Ezh2^R684C/+^* DMSO versus *Ezh2^+/+^* DMSO contrast. Blue: MGI osteoblast differentiation genes. Magenta: MGI BMP pathway genes. FDR = 0.1 (red dashed line). (**E**) Conditional *P* value density plot displaying *P* values from the day 14 untreated RNA-Seq, stratified by significance at day 21 in the *Ezh2^R684C/+^* DMSO versus *Ezh2^+/+^* DMSO contrast (orange line, *P*_adj_ < 0.1) or not (black line, *P*_adj_ > 0.1). (**F**) Principal component analysis of *Ezh2^+/+^* (blue circles, *n* = 6) and *Ezh2^R684C/+^* (red triangles, *n* = 6) RNA-Seq samples at day 21 of osteoblast differentiation, treated either with vehicle (filled icons) or GSK-J4 (open icons). Corresponding Alizarin red staining images shown for representative samples. (**G**) Volcano plot of day 21 RNA-Seq, displaying log_2_ fold changes in the *Ezh2^R684C/+^* GSK-J4 versus *Ezh2^R684C/+^* DMSO contrast. FDR = 0.1 (red dashed line). (**H**) Scatter plot comparing log_2_ fold changes in the *Ezh2^R684C/+^* DMSO versus *Ezh2^+/+^* DMSO contrast and corresponding log_2_ fold-changes in the *Ezh2^R684C/+^* GSK-J4 versus *Ezh2^R684C/+^* DMSO contrast. Only genes meeting an adjusted *P* value threshold corresponding to FDR < 0.1 in both contrasts are shown (*n* = 1,075). Blue: MGI osteoblast differentiation genes. Magenta: MGI BMP pathway genes.

Because EZH2 is the primary methyltransferase (writer) of H3K27me3, we reasoned that the characteristic features of Weaver syndrome might be attributed to decreased or displaced H3K27me3 at key genomic loci. A potential therapeutic strategy, therefore, could involve rebalancing the epigenome through inhibition of the opposing H3K27me3 demethylases (erasers) (Figure 5A). We treated female *Ezh2^R684C/+^* and *Ezh2^+/+^* BM-MSCs with either DMSO (vehicle) or GSK-J4, which is a dual inhibitor of KDM6A and KDM6B (35), the primary H3K27me3 erasers in mice and humans (36, 37). Vehicle-treated *Ezh2^R684C/+^* cells had appreciably increased Alizarin red staining after 21 days of osteoblast differentiation compared with vehicle-treated *Ezh2^+/+^* cells (adjusted *P* [*P*_adj_] = 0.006) (Figure 5, B and C), reinforcing our earlier finding in untreated osteoblasts (Figure 3, D and E). For *Ezh2^R684C/+^*, cells treated with 2 μM of GSK-J4 during days 0–7 of differentiation had notably decreased Alizarin red staining compared with vehicle-treated cells (*P*_adj_ = 0.030) (Figure 5, B and C). There was no observed decrease in cell viability due to GSK-J4 treatment at 2 μM (Supplemental Figure 6, A and B). Importantly, GSK-J4–treated *Ezh2^R684C/+^* cells showed no statistically significant difference in Alizarin red staining compared with DMSO-treated *Ezh2^+/+^* cells (*P*_adj_ = 0.929). This suggests that GSK-J4 can reverse the excessive osteogenesis of *Ezh2^R684C/+^* osteoblasts toward the WT baseline. Similar results were found in male *Ezh2^R684C/+^* and *Ezh2^+/+^* osteoblasts (Supplemental Figure 6, C and D).

### GSK-J4 alters the transcriptional profile of in vitro differentiated Ezh2^R684C/+^ osteoblasts.

To determine the molecular basis of the GSK-J4 therapeutic effect, we investigated whether GSK-J4 could reverse the transcriptional state of *Ezh2^R684C/+^* cells toward a WT profile. To this end, we performed RNA-Seq on *Ezh2^R684C/+^* and *Ezh2^+/+^* osteoblasts. Cells from 6 female mice per genotype were treated with either DMSO (vehicle) or 2 μM of GSK-J4 for the first 7 days of differentiation and harvested on day 21. Comparison of vehicle-treated *Ezh2^R684C/+^* (*R684C/+* DMSO) to vehicle-treated *Ezh2^+/+^* (*+/+* DMSO) osteoblasts yielded 2,659 DEGs at the 10% FDR level (Figure 5D) (Supplemental Appendix 4). This is more than was observed between genotypes in our earlier RNA-Seq on day 14 untreated cells (Supplemental Figure 4A) and is not accounted for by differences in mean read counts per gene (Supplemental Figure 7A) or coefficients of variation (Supplemental Figure 7, B–D). Reassuringly, among the abundance of significant hits in the *R684C/+* DMSO versus *+/+* DMSO comparison, there was a clear enrichment of genes previously identified as differentially expressed at day 14 (Figure 5E). Based on these results, we surmise that, between day 14 and day 21, when the final osteoblast differentiation state is achieved, genotype-specific effects create a greater differential in transcriptional profile between *Ezh2^R684C/+^* and *Ezh2^+/+^* cells.

Principal component analysis of all samples showed that PC1 accounted for 77% of gene expression variance and was closely correlated with corresponding Alizarin red quantifications for the same cell line and treatment condition (*r* = –0.89) (Figure 5F). To verify that the RNA-Seq data were showing an osteogenic transcriptional signal, we also ran linear correlations between Alizarin red quantifications and individual gene CPM. Among the 20 genes with the highest positive correlation coefficients were *Col1a1*, *Col1a2*, *Smad3*, and *Cthrc1*, indicating that the RNA-Seq captured an osteogenic transcriptional profile in samples with high uptake of Alizarin red (38–40).

The *R684C/+* DMSO versus *+/+* DMSO contrast represents genes that are perturbed by the R684C allele. An ideal therapeutic would reverse the transcriptional effects of R684C while minimizing disturbance to the rest of the transcriptome, although this efficacy and specificity is difficult to achieve in practice. The effect of GSK-J4 upon the *Ezh2^R684C/+^* phenotype is captured by comparing GSK-J4–treated *Ezh2^R684C/+^* cells (*R684C/+* GSK-J4) with vehicle-treated *Ezh2^R684C/+^* cells (*R684C/+* DMSO). This contrast yielded an upregulation of 1,562 genes in the *R684C/+* GSK-J4 condition and a downregulation of 1,484 genes, for a total of 3,046 DEGs at the 10% FDR level (Figure 5G) (Supplemental Appendix 5). Of these, 1,075 genes were also differentially expressed in the *R684C/+* DMSO versus *+/+* DMSO contrast — i.e., both perturbed by the R684C allele and targeted by GSK-J4. Most remarkably, 1,045 of the 1,075 shared genes appeared to reverse their fold-change directionality with GSK-J4 treatment (Figure 5H), including 19 genes in the BMP pathway and 31 genes involved in osteoblast differentiation (8 of these overlap). For example, *Satb2*, *Runx2*, *Dlx3*, and *Pthr1* are upregulated in the *R684C/+* DMSO condition relative to *+/+* DMSO, but they become downregulated upon GSK-J4 treatment (Figure 5, D and G). These results indicate that GSK-J4 is able to reverse much of the altered transcriptional profile of *Ezh2^R684C/+^* cells toward a WT state.

To determine whether the transcriptional alterations in *Ezh2^R684C/+^* cells and reversal upon GSK-J4 treatment are a direct effect of changes in H3K27me3 levels, we performed ChIP-qPCR. We differentiated *Ezh2^+/+^* and *Ezh2^R684C/+^* cells toward osteoblasts for 21 days, with either DMSO or GSK-J4 treatment, and performed immunoprecipitation of the genomic DNA with an anti-H3K27me3 antibody. Interestingly, we did not see any significant genotype- or treatment-based differences in H3K27me3 enrichment at the promoter regions of *Satb2*, *Runx2*, or *Pth1r* (Supplemental Figure 8, A–G). This suggested that these 3 particular loci may not be direct targets of EZH2.

## Discussion

Here we developed and characterized what we believe to be a novel mouse model for the most commonly encountered pathogenic variant in Weaver syndrome, EZH2 p.R684C. Individuals with Weaver syndrome are often tall, with heights > 2 SDs above the age-matched mean, and have additional skeletal abnormalities such as advanced osseous maturation and metaphyseal widening (13, 14, 16). *Ezh2^R684C/+^* mice display skeletal overgrowth and bone structural abnormalities, which are reminiscent of Weaver syndrome. We show this overgrowth to be driven by hyperactivity of the osteoblast lineage in mice. In addition, BM-MSCs isolated from *Ezh2^R684C/+^* mice undergo excessive osteogenesis upon and even prior to differentiation, and this was reflected both at the levels of the transcriptome and the cellular phenotype. This may underlie the skeletal overgrowth and advanced bone age observed in many individuals with Weaver syndrome.

### Overgrowth is more subtle in mouse models of Weaver syndrome than in human individuals.

While extreme tall stature is a frequent manifestation of Weaver syndrome, with some individuals achieving heights up to 7.6 SD or more above the age-matched population mean (14, 16), this feature is less prominent in *Ezh2^R684C/+^* mice. We did not observe a significant difference in femur, tibia, or total body length compared with *Ezh2^+/+^* littermates, and only female *Ezh2^R684C/+^* mice had increased weight at 8 weeks. Our results are consistent with those observed in a mouse model for another Weaver syndrome missense variant, V626M (41). In these *Ezh2^V626M/+^* mice, tibia length was also unchanged, and there was a sexually dimorphic effect on weight, with female *Ezh2^V626M/+^* mice having a greater increase in weight. Overgrowth has not been reported as more pronounced in female individuals with Weaver syndrome, although this may be due to a lack of systematic investigation, since reported cases of Weaver syndrome number < 100.

The majority of MDEMs have growth abnormalities (2), and epigenetic machinery (EM) genes are overrepresented as causes of overgrowth (42). This implies that genes involved in skeletal growth may be under a greater degree of control by epigenetic mechanisms and, thus, particularly vulnerable to imbalances in the EM. However, it has been challenging to model overgrowth of MDEMs in mice. The first heterozygous *Ezh2*-KO mouse model appeared to have no phenotype, although in-depth skeletal profiling was not performed (43). Sotos syndrome (MIM 117550) is by far the most common MDEM with overgrowth (42), caused by pathogenic variants in *NSD1* as well as 5q35 microdeletions that span *NSD1* (44). However, mice heterozygous for the syntenic microdeletion were found to have decreased weight in utero and at 28 weeks of age (45). *Nsd1^+/–^* mice, with loss of 1 allele due to a premature termination codon (PTC), display some characteristic behavioral abnormalities but do not have overt differences in body weight (46). More detailed profiling with μ-CT may nevertheless shed light on features of skeletal overgrowth, as in our *Ezh2^R684C/+^* mice. Transgenic mice are invaluable tools in elucidating the mechanisms of rare disorders; however, it is also important to remain cognizant of potential differences in skeletal growth regulation between mice and humans.

### R684C appears to be a loss-of-function/hypomorphic variant.

The mechanism by which *EZH2* variants cause Weaver syndrome has been a matter of discussion. Almost all patients bear heterozygous missense variants (14–16, 42). Cohen et al. previously showed in vitro that EZH2 p.R684C, as well as several other missense variants reported in Weaver syndrome, had reduced incorporation of ^3^H-S-adenosyl-methionine (^3^H-SAM) onto core histones (47). We showed that neither *Ezh2^R684C/R684C^* nor *Ezh2^R684C/+^* MEFs have decreased EZH2 protein levels, yet we observed that *Ezh2^R684C/R684C^* MEFs experience a drastic loss of H3K27me3. Importantly, we also showed that global H3K27me3 levels in *Ezh2^R684C/+^* MEFs are intermediate with respect to *Ezh2^+/+^* and *Ezh2^R684C/R684C^* MEFs; this supports a mechanism of loss-of-function or hypomorphic activity by the *R684C* allele and is in agreement with Cohen et al. (47). This concurrently rules out the possibility of paradoxical hypermorphic activity, which is known to occur with certain somatic *EZH2* variants found in B cell lymphomas (48, 49).

In contrast, a recent study instead suggested that R684C could have a dominant negative mechanism. Deevy et al. used lentiviral transduction to overexpress EZH2 p.R684C, as well as other Weaver syndrome missense variants, in mouse embryonic stem cells (ESCs) that were heterozygous KO for *Ezh2* (*Ezh2*^fl/Δ^) (50). The global decrease in H3K27me3 resulting from the missense variants was notably more severe than in nontransduced *Ezh2*^fl/Δ^, which was corroborated by H3K27me3 ChIP-Seq. Discrepancies between our findings and those of Deevy et al. on the R684C variant may be due to differences in our model systems, as we used primary MEFs isolated from *Ezh2^R684C/+^* mice at a considerably later developmental stage than ESCs. In the future, it would be interesting to investigate loci-specific H3K27me3 occupancy, as well, in our system and especially in osteoblasts.

### Haploinsufficient variants are underrepresented in Weaver syndrome.

While most MDEMs result from haploinsufficiency (2), the extreme preponderance of missense variants in Weaver syndrome over frameshift, nonsense, and whole-gene deletion variants suggests an alternate mechanism. One possibility is that manifestation of the Weaver phenotype may depend upon maintaining normal levels of EZH2 protein production, even if reduced in catalytic capacity. Proteins frequently bear multiple functions, known as “moonlighting” (51). EZH2 is known for its H3K27 methyltransferase activity, but it may also play a functional role independent of catalysis. Only 4 EZH2 variants leading to PTCs have been published — all within the last exon and therefore predicted to escape nonsense-mediated decay (NMD) and result in a protein product (16). More recently, 4 nonsense and 3 frameshift variants that would be expected to result in NMD were uploaded to ClinVar (52), but it remains to be seen whether a reduction in EZH2 protein leads to the same phenotype as Weaver missense variants.

Since heterozygous whole-gene deletions can be assumed to reduce expression of the corresponding protein, we scanned for deletions encompassing *EZH2* in DECIPHER, a database linking human genetic variation with phenotype. Interestingly, of the 78 *EZH2* heterozygous whole-gene deletion variants with corresponding phenotype documentation available in DECIPHER, none are associated with overgrowth or tall stature (53). Rather, the second most common phenotype annotation is short stature, seen in 29 individuals (the top phenotype is intellectual disability, in 39 individuals). Since these deletions range from 5.10 Mb to 27.36 Mb in length and span multiple genes, phenotypic effects cannot be attributed solely to the loss of *EZH2*. Indeed, a patient bearing a relatively smaller 1.2 Mb deletion has been reported to exhibit features of Weaver syndrome, such as tall stature, intellectual disability, and select facial dysmorphisms, which supports a mechanism of haploinsufficiency (54). However, the possibility remains that *EZH2* haploinsufficiency in humans could constitute a distinct phenotype from missense variants. Without clinical suspicion for Weaver syndrome, such variants may escape detection, leading to underreporting.

### Both direct and indirect transcriptional effects may drive MDEM phenotypes.

EM genes are dosage sensitive (55), and EZH2 is the predominant mammalian methyltransferase for the transcriptionally silencing H3K27me3 mark. Therefore, we originally hypothesized that decreased EZH2 activity would lead to widespread gene derepression. However, there is no skew toward upregulation of gene expression in *Ezh2^R684C/+^* cells, suggesting that derepressed EZH2 target genes may be drowned out by innumerable downstream indirect effects. Despite the statistical significance of these gene expression changes, many of the corresponding fold-changes in expression were also relatively modest. In agreement with prior literature on MDEMs (6), we view these findings in light of the Boyle et al. “omnigenic” model, which supposes that genes are highly interconnected regulatory networks (56). Perturbation of a few core genes, which have a direct biological link to disease, leads to small regulatory changes in a large number of interconnected genes. This is also concordant with our observation that, of all the genes with altered expression, the great majority were not directly implicated in the osteogenic phenotype. The transcriptional profile of MDEMs such as Weaver syndrome can, thus, be thought of as the sum of direct effects due to locus-specific epigenetic modification as well as consequent indirect effects upon the gene regulatory network.

In an attempt to discern whether the transcriptional effects we observed in *Ezh2^R684C/+^* cells and upon GSK-J4 treatment were due to direct EZH2 activity, we performed H3K27me3 ChIP-qPCR at several genes of interest. For this limited selection of loci, H3K27me3 levels remained unaltered in *Ezh2^R684C/+^* cells and also with drug treatment, suggesting that they are not direct targets of EZH2. However, given the significant decrease in H3K27me3 seen globally, we predict that this mark may be altered at other loci that are not captured by our short-range ChIP-qPCR probes. Although outside the scope of this paper, H3K27me3 ChIP-Seq would allow genome-wide profiling of the levels of this mark, thus providing a more comprehensive answer. An even more ideal alternative would be to perform ChIP-Seq for EZH2, revealing its direct binding sites across the genome under various conditions.

Another possible route through which EZH2 may exert indirect effects is methylation of nonhistone substrates such as the nuclear receptor RORα (57). Through this capacity, EZH2 involvement has been demonstrated in methylation-dependent ubiquitination and proteasomal degradation pathways. Such alternative catalytic activity need not be mutually exclusive with the role of EZH2 in histone methylation, and it is conceivable that both contribute toward the phenotype of Weaver syndrome. While prior studies and ours do not delve into this, it remains an intriguing possibility that may be worth exploration in the future.

### Abnormal cell differentiation is a shared feature of MDEMs.

Cell fate decisions are highly regulated processes in multicellular organisms. Epigenetic control of cell type–specific programs is critical to achieve spatiotemporally appropriate differentiation, and loss of this control has disastrous consequences (58). It is therefore unsurprising that the epigenomic disruption caused by MDEMs affects cell differentiation. Our results in *Ezh2^R684C/+^* mice suggest that, in Weaver syndrome, osteoblast differentiation is perturbed, at least in part through dysregulation of the BMP pathway. Excessive osteogenesis is concordant with the human phenotype of skeletal overgrowth and advanced osseous maturation. Studies of other MDEMs also indicate aberrant differentiation in phenotype-relevant tissues: precocious differentiation of chondrocytes and neural stem and progenitor cells (NSPCs) in Kabuki syndrome 1 (4, 5), and delayed maturation of NSPCs in Rubinstein-Taybi syndrome (MIM 180849) (59). Further investigation of other relevant cell types in Weaver syndrome may yield additional findings of altered differentiation.

### Epigenetic modifying agents such as GSK-J4 represent a promising approach to treating MDEMs.

No targeted therapies are yet approved for use in MDEMs, but advances are being made. Currently, MDEMs including Weaver syndrome are managed in clinic on the basis of ameliorating symptoms, which are often multisystemic (3). Here, we sought a treatment to address the mechanistic root of Weaver syndrome. Fahrner and Björnsson previously proposed the “balance hypothesis,” stating that opposing writers and erasers of histone marks exist in a balance to maintain the normal chromatin state (1). MDEMs are thought to disrupt this balance by inappropriately increasing or reducing levels of epigenetic modifications at target loci, leading to an abnormal chromatin state. Several groups have adopted a strategy of using epigenetic modifying agents to restore balance in MDEMs, specifically Rubinstein-Taybi syndrome and Kabuki syndrome 1 (60–63). Alarcón et al. first demonstrated that cognitive defects in a mouse model of Rubenstein-Taybi could be ameliorated by directly counteracting haploinsufficiency of the histone acetyltransferase, CBP, with histone deacetylase inhibitor (HDACi) (60). In the case of Kabuki syndrome 1, which is predicted to have loss of the transcriptionally activating mark H3K4me3 at promoters, the Björnsson group showed that either inhibition of the opposing H3K4 demethylase (63) or HDAC inhibition through pharmacological or diet-induced means was also sufficient to improve hippocampal function and visual-spatial learning in mice (61, 62). Therefore, correction of overall chromatin state may prove just as impactful as restoration of a specific epigenetic mark, albeit at a risk of lower specificity.

While the above studies focus on treatment of neurological features, our findings suggest that a similar approach also has the potential to prevent or reverse skeletal overgrowth, another common feature of MDEMs. Here, we tested the ability of GSK-J4, an inhibitor of lysine demethylases KDM6A and KDM6B, to counteract the loss of EZH2 function. The balance between the primary writer and erasers of H3K27me3 has previously been implicated in human bone biology (64, 65). Inhibition of EZH2 by GSK-126, a pharmacological analogy to Weaver syndrome, stimulates osteoblast differentiation in mice (66), while GSK-J4 reduced osteogenesis in a mouse model of Saethre-Chotzen craniosynostosis (MIM 101400) (67).

We showed that GSK-J4 not only reduces mineralization by *Ezh2^R684C/+^* BM-MSCs toward WT, but it also substantially reverses effects of the variant allele at the transcriptional level. Of note, we previously found that correcting the expression of a single target gene was insufficient to restore normal chondrogenesis in Kabuki syndrome 1 (5). Since KDM6A and KDM6B act broadly across the genome, exerting both direct and indirect transcriptional regulation, the precise restoration of the WT gene expression profile by GSK-J4 is an improbable goal. From a statistical perspective, there are downsides to comparing GSK-J4–treated *Ezh2^R684C/+^* osteoblasts with vehicle-treated *Ezh2^+/+^* control osteoblasts directly. This would entail establishing a lack of significant difference, a task potentially confounded by unequal intersample variances between the groups. Rather, our analysis (comparing GSK-J4–treated *Ezh2^R684C/+^* with vehicle-treated *Ezh2^R684C/+^* and comparing vehicle-treated *Ezh2^R684C/+^* with vehicle-treated *Ezh2^+/+^*) looked for general reversal of fold-change directionality upon GSK-J4 treatment among genes found to be dysregulated in *Ezh2^R684C/+^* osteoblasts. We showed that the transcriptional effect of GSK-J4 involved a fold-change reversal of more than 1,000 genes, suggesting that a large network of pathogenic gene expression may need to be addressed in order to achieve phenotypic rescue in MDEMs. This finding stresses the importance of developing epigenetic modifying agents to treat MDEMs, since these drugs hold the prospect of enacting the broad transcriptomic changes required across a multitude of tissues. Our results constitute what we believe to be the first demonstration of using an epigenetic modifying agent to treat growth abnormalities in MDEMs, and they strongly support the potential of GSK-J4 as a therapeutic for Weaver syndrome and possibly other overgrowth disorders impacting PRC2 function.

### Conclusion.

Using our *Ezh2^R684C/+^* mouse model, we showed that osteoblasts are a key cell type contributing toward skeletal overgrowth in Weaver syndrome. We reversed the pathological osteogenic phenotype of *Ezh2^R684C/+^* osteoblasts in vitro with GSK-J4, an epigenetic modifying agent targeting KDM6A/KDM6B, the demethylases complementary to EZH2. Future studies on the locus-specific role of H3K27 methylation in growth regulation are called for to shed light on molecular mechanisms of Weaver syndrome and related PRC2 syndromes. The ultimate goal of this work is to contribute toward the development of therapeutics to target the underlying etiology of MDEMs as a class of disorders.

## Methods

### Animals.

*Ezh2^R684C/+^* mice were generated by the Johns Hopkins Transgenic Core Laboratory using CRISPR-Cas9 gene editing. The *Ezh2*-specific guide RNA (5′-GTGGTGGATGCAACCCGAAA-3′) and the homology-directed repair (HDR) template (5′-ACTGAAAATAAGTCACTGGATTATCTATGTTTTTCACTTTAGATTTTGTGGTGG ATGCAACatGtAAaGGCAACAAAATTCGTTTTGCTAATCATTCAGTAAATCCAAACTGCTATGCAAAAGGTA-3′) were based on mouse transcript NM_007971.2/ ENSMUST00000081721.12. The HDR template included mutations (indicated in lowercase) necessary to generate the mouse p.R679C missense change, which corresponds to human p.R684C. In addition, silent base changes were introduced to engineer an Nsp1 restriction site for rapid genotyping. C57BL/6J embryos were injected with Cas9 mRNA, the gRNA, and the HDR DNA oligo in the presence of SCR7. One founder was obtained and crossed to C57BL/6J, demonstrating germline transmission. Mice were backcrossed for 9 generations prior to experimental use in order to remove potential off-target sequence changes and were maintained on a C57BL/6J background (strain no. 000664, The Jackson Laboratory). Genotyping was performed by PCR and Sanger sequencing, in some cases accompanied by Nsp1 digest. Mice were cared for by Johns Hopkins Research Animal Resources. Mice were housed up to 5 per barrier cage in ventilated racks with HEPA-filtered and humidified air, under standardized light/dark cycles. Mice had ad libitum access to autoclaved feed (Envigo Teklad, 2018SX) and reverse osmosis-filtered, hyperchlorinated water. Each cage was provided with autoclaved corncob bedding (Envigo Teklad, 7092/7097) and a cotton square nestlet (Envigo Teklad, 6060/6105). Cages were changed every 2 weeks under aseptic conditions. Euthanasia was performed by halothane inhalation (Sigma-Aldrich, B4388), following the AVMA Guidelines for the Euthanasia of Animals, 2020 edition (https://olaw.nih.gov/policies-laws/avma-guidelines-2020.htm).

### MEFs.

*Ezh2^R684C/+^* female mice underwent timed matings with *Ezh2^R684C/+^* male mice. Pregnant females were euthanized at E14.5. Embryos were dissected from the uteri; minced in DMEM (Thermo Fisher Scientific, 11960044) with 15% FBS (Thermo Fisher Scientific, 16140071), L-glutamine (Corning, 25-005-CI), nonessential amino acids (Thermo Fisher Scientific, 11140076), and penicillin/streptomycin (Thermo Fisher Scientific, 15140122); and dissociated with gentle pipetting. Cell suspension was cultured in DMEM (Thermo Fisher Scientific, 11960044) with 10% FBS (Thermo Fisher Scientific, 16140071), L-glutamine (Corning, 25-005-CI), nonessential amino acids (Thermo Fisher Scientific, 11140076), and penicillin/streptomycin (Thermo Fisher Scientific, 15140122) at 37°C, 5% CO_2_, with media changes every 48–72 hours.

### Mouse BM-MSCs.

Mice were euthanized at 8 or 10 weeks of age (males or females, respectively). Femora and tibiae were dissected immediately and rinsed with penicillin/streptomycin (Thermo Fisher Scientific, 15140122) in phosphate-buffered saline. BM was flushed out of the medullary cavity with BM-MSC complete growth media, consisting of MEM Alpha (Corning, 10-022-CV) supplemented with 15% heat-inactivated FBS (Thermo Fisher Scientific, 16140071), 2 mM L-glutamine (Corning, 25-005-CI), and 100 U/mL penicillin and 100 μg/mL streptomycin (Thermo Fisher Scientific, 15140122). BM was passed through a 70 μm cell strainer and cultured for 2 weeks in BM-MSC complete growth media at 37°C and 5% CO_2_. Media were refreshed every 48–72 hours. Adherent BM-MSCs were dissociated with 0.25% trypsin and 2.21 mM EDTA (Corning 25-053-CL) and were seeded for subsequent experiments.

### Osteogenic differentiation.

BM-MSCs were seeded at a density of 1 × 10^5^ cells/cm^2^ in BM-MSC complete growth media. After 24 hours, complete media growth was swapped for osteogenic differentiation media. Osteogenic differentiation media consisted of BM-MSC complete growth media supplemented with 0.05 mg/mL L-ascorbic acid (Sigma-Aldrich, A4403), 10 mM β-glycerophosphoric acid (Thermo Fisher Scientific, 410991000), and 102 nM dexamethasone (Sigma-Aldrich, D4902). Cells were continually cultured for 21 days at 37°C and 5% CO_2_. Osteogenic differentiation media were refreshed every 4872 hours.

### Western blot.

MEFs were washed twice with cold 1× PBS and lysed using RIPA buffer with a protease/phosphatase inhibitor cocktail (Cell Signaling Technology, 5872) to obtain total protein samples. Histones were isolated with a Histone Extraction kit (Abcam, ab113476). Protein concentration was assessed with the Pierce BCA Protein Assay kit (Thermo Fisher Scientific, 23225), and 20 μg of each sample was loaded onto precast NuPAGE 4%–12%, Bis-Tris gels (Thermo Fisher Scientific, NP0336BOX). Proteins were transferred to a PVDF membrane. Intercept (PBS) Blocking Buffer (0.5×, LI-COR Biosciences 927-70001) was used to block the membrane for 1 hour at room temperature. Membranes were incubated with the primary antibody overnight at 4°C and were then washed using 1× PBS with 0.1% Tween-20 (PBS-T). Secondary antibody was added for 1 hour at room temperature. PBS-T was used to wash off unbound secondary antibody. Membranes were imaged with the LI-COR Odyssey. Quantification was performed using ImageJ software (NIH). The optical density of EZH2 bands was normalized to ACTB, and H3K27me3 was normalized to H3 after adjusting for background. All Western blot antibodies are listed in Supplemental Table 2A.

### High-resolution μ-CT.

Femurs and tibias were dissected from 8-week-old mice and fixed with 4% paraformaldehyde in 1× PBS for 48–72 hours before transfer to 70% ethanol. Bone length was assessed using digital calipers before high-resolution images were obtained with a Bruker Skyscan 1275 desktop μ-CT system. Long bones were scanned at 65 keV and 152 μA using a 0.5 mm aluminum filter at an isotropic voxel size of 10 μm, in accordance with the recommendations of the American Society for Bone and Mineral Research (ASBMR) (68). μ-CT images were reconstructed with nRecon (Bruker) and analyzed with CTAn 3D analysis software (Bruker). Cortical bone structure was assessed in a 500 μm region of interest (ROI) centered on the femoral middiaphysis. Trabecular bone structure was assessed in a 2 mm ROI located 500 μm proximal to the distal femoral growth plate.

### Dynamic bone histomorphometry.

Five-week-old mice were injected i.p. with 10 mg/kg of calcein (Sigma-Aldrich, C0875) dissolved in a 2% sodium bicarbonate solution, 5 days prior to euthanasia. Mice were then injected with 30 mg/kg of Alizarin red S (Sigma-Aldrich, A3882) dissolved in a 2% sodium bicarbonate solution, 2 days prior to euthanasia. After collection, femurs were fixed in 100% ethanol until embedding in methyl methacrylate. Sections of 30 μm were obtained from the femoral middiaphysis and visualized with fluorescence microscopy. Histological analyses were performed using ImageJ in accordance with the ASBMR guidelines. Images were analyzed for Es and Ps MAR, as defined by the ASBMR Committee for Histomorphometry Nomenclature (69, 70).

### Alizarin red staining.

After 21 days of osteogenic differentiation, cells were washed with 1× PBS and fixed with 10% neutral buffered formalin (Epredia, 9990244) for up to 1 hour. Following fixation, cells were washed with deionized water and stained with 2% Alizarin red S solution, pH 4.2 (Electron Microscopy Sciences 26206-01), for 1 hour in the dark. Unbound dye was removed by repeated washes with deionized water. Whole-well images were taken over a transilluminator without magnification. The following protocol for quantification of Alizarin red was adapted from Serguienko et al. (71). Dye bound by the calcified matrix was dissolved by incubation in 10% acetic acid for 30 minutes with gentle agitation. Cells were scraped and transferred with the acetic acid solution to polypropylene tubes. Samples were heated to 85°C with agitation for 10 minutes, rapid-cooled on ice, and then pelleted at 20,000*g*. Supernatant was retained and neutralized with 10% ammonium hydroxide to pH 4.1–4.5. Absorbance was measured at 405 nm with a BioTek Synergy 2 Multi-Mode Microplate Reader.

### MTT assay.

CellTiter 96 AQueous One Solution (Promega, G3580) was added to fresh BM-MSC complete growth media. Cells were incubated in the dark at 37°C, 5% CO_2_, for 1.5 hours to allow for conversion to formazan. Absorbance was measured at 490 nm with a BioTek Synergy 2 Multi-Mode Microplate Reader.

### qPCR.

Total RNA was isolated using TRIzol reagent (Thermo Fisher Scientific, 15596026). The SuperScript IV First-Strand Synthesis system (Thermo Fisher Scientific, 18091050) was used to reverse transcribe the total RNA into cDNA. qPCR was performed with the PowerUp SYBR Green Master Mix (Thermo Fisher Scientific, A25742) on an Applied Biosystems ViiA 7 Real-Time PCR System. Primers are listed in Supplemental Table 2B.

### GSK-J4 treatment.

GSK-J4 crystalline solid (Cayman, 12073) was reconstituted in dimethyl sulfoxide (Sigma-Aldrich, D2650) to a stock concentration of 10 mM. BM-MSCs were seeded at a density of 1 × 10^5^ cells/cm^2^ in BM-MSC complete growth media. After 24 hours, this was replaced by osteogenic differentiation media containing either GSK-J4 (treatment group) or the equivalent volume of DMSO (vehicle group). DMSO content did not exceed 0.1% of media volume for the highest treatment dose concentration. Cells were treated for 7 days, after which culture was maintained in osteogenic differentiation media without GSK-J4 or DMSO until day 21. Media were exchanged every 48–72 hours.

### RNA-Seq library preparation.

BM-MSCs were isolated from 6 mice each for *Ezh2^R684C/+^* and *Ezh2^+/+^* genotypes. BM-MSCs underwent osteogenic differentiation for 14 or 21 days. For the latter experiment, cells were treated with 2 μM of GSK-J4 or equivalent volume of DMSO (GSK-J4 treatment). Cells were lysed and homogenized with TRIzol reagent (Thermo Fisher Scientific, 15596026), followed by phenol-chloroform separation. Total RNA was purified from the aqueous phase using the RNA Clean & Concentrator-5 kit with DNase I treatment to remove genomic DNA contamination (Zymo Research, R1013). RNA quantity was determined with the Qubit RNA Broad Range Assay (Thermo Fisher Scientific, Q10210). RNA quality was assessed with the Agilent RNA 6000 Nano Kit (Agilent, 5067-1511) run on an Agilent 2100 Bioanalyzer instrument or submitted to the Johns Hopkins Single Cell & Transcriptomics Core (JH SCTC) Facility to be processed on an Agilent Fragment Analyzer. Polyadenylated RNA was isolated from either 1 μg (day 14) or 300 ng (day 21) of total RNA using the NEBNext Poly(A) mRNA Magnetic Isolation Module (New England BioLabs, E7490L). Libraries were prepared using the NEBNext Ultra II RNA Library Prep kit with Sample Purification Beads (New England BioLabs, E7775S) and indexed with NEBNext Multiplex Oligos for Illumina (Dual Index Primers Set 1) (New England BioLabs, E7600S). Library quality was assessed with the Agilent High Sensitivity DNA Kit (Agilent, 5067-4626) run on the Agilent 2100 Bioanalyzer instrument or submitted to the JH SCTC for processing on an Agilent Fragment Analyzer. Completed libraries were quantified using the NEBNext Library Quant Kit for Illumina (New England BioLabs, E7630L) and pooled accordingly to a final concentration of 4 nM. High-throughput sequencing was performed by the Johns Hopkins Genomics Research Core Facility on the Illumina NovaSeq 6000 platform using SP flow cells to generate 100 bp paired-end reads.

### Analysis of RNA-Seq data.

Salmon 1.9.0 was used to index the GRCm38 transcriptome (72), using the GRCm38 genome as the decoy sequence; both are available through Ensembl (Mus_musculus.GRCm38.cdna.all.fa.gz and Mus_musculus.GRCm38.dna.primary_assembly.fa.gz, from http://nov2020.archive.ensembl.org/Mus_musculus/Info/Index, release 102). Lane read outputs were demultiplexed into raw FASTQ files for each sample, which were mapped using Salmon 1.9.0 for paired-end reads, with selective alignment and GC bias correction enabled. Transcript quantifications were imported into R 4.1.2, running Bioconductor 3.14 and summarized to gene-level counts using tximeta 1.12.4 (73). Gene counts for any technical replicates were combined, as principal component analyses indicated minimal difference between technical replicates. Non- and low-expressed genes (median count across all samples < 10) were filtered. Surrogate variables (SVs) were identified and accounted for using sva 3.42.0 (2 SVs for D14; 4 SVs for D21) (74). DESeq2 1.34.0 was used to perform differential expression analysis (75). A FDR cutoff < 0.1 was used to determine significance; the default DESeq2 correction for multiple comparisons is the Benjamini-Hochberg procedure. Genes were subsequently annotated with biomaRt 2.50.3, accessing version 102 of the *Mus musculus* Ensembl data set (76, 77). Lists of DEGs are available (Supplemental Appendices 1, 4, and 5). The MGI database was accessed on March 25, 2023, to download lists of genes with the GO annotations “osteoblast differentiation” and “BMP signaling pathway” (Supplemental Appendices 2 and 3). Two-sample Wilcoxon test statistics were calculated for the *P* values of genes falling within and outside of the annotated subsets. These were compared with the Wilcoxon test statistic distribution calculated for 10,000 gene subsets of equal length, chosen at random.

### Validation of RNA-Seq data using an external dataset.

Raw read FASTQ files were downloaded from GEO (accession no. GSE138980; ref. 34). Samples treated with the EZH2 inhibitor GSK-126 were compared with samples treated with nontargeting siRNA (control group). Read mapping and differential expression analysis was performed as described above, maintaining a FDR cutoff < 0.1. A conditional *P* value histogram was generated to examine the relative enrichment of DEGs in *Ezh2^R684C/+^* early osteoblasts among the pool of DEGs resulting from pharmacological inhibition of EZH2 in murine MSCs.

### Determination of EZH2 target genes.

The “Transcription Factor ChIP-Seq Clusters (161 factors) from ENCODE with Factorbook Motifs’” track data was downloaded from the UCSC Genome Browser (wgEncodeRegTfbsClusteredWithCellsV3.bed.gz, http://hgdownload.soe.ucsc.edu/goldenPath/hg19/encodeDCC/wgEncodeRegTfbsClustered/) (78–82). EZH2-bound clusters were selected and converted to a GRanges object using GenomicRanges 1.46.1 (83). The locations of all hg19 gene promoters (± 2 kb of the transcription start site) were determined using the reference genome package BSgenome.Hsapiens.UCSC.hg19 alongside the annotation package EnsDb.Hsapiens.v75. A gene was considered an EZH2 target if its promoter overlapped with an EZH2-bound cluster. biomaRt 2.50.3 was used to match hg19 EZH2 target genes with mouse homologs (76, 77).

### ChIP-qPCR.

BM-MSCs were seeded in 6 cm dishes at 2 × 10^6^ cells per dish. Cells were treated with either GSK-J4 or DMSO as described above and differentiated toward osteoblasts for 21 days. ChIP protocol was adapted from Fang et al. (84). Refer to Fang et al. for buffer recipes. In brief, cells were fixed with 16% formaldehyde for 10 minutes, followed by neutralization with glycine. After washing with cold TBS, cell lysis buffer was added. Lysates were harvested and pelleted. Chromatin was digested with MNase (New England Biolabs, M0247S) for 20 minutes at 37°C shaking at 1,000 rpm. Further fragmentation was achieved by sonication with the Diagenode Bioruptor Plus for 20 minutes with 30-second on/off cycles. Chromatin concentration was assessed with the Qubit DNA High Sensitivity kit. In total, 1 μg of chromatin was used per IP reaction with 5 μg of anti-H3K27me3 antibody (Active Motif, 61018, clone MABI 0323, lot no. 15819018). Prior to addition of antibody, a 4% Input sample was collected and stored at –80°C. IP reactions were rotated overnight at 4°C. The following day (day 2), reactions were incubated with Protein G Magnetic Sepharose Xtra beads (Cytiva, 28-9670-66) for 3 hours at 4°C to pull down antibody-bound chromatin. Beads were washed extensively with ChIP Buffer, High Salt Buffer, Tris/LiCl_2_ Buffer, and TE Buffer. Bound fraction was eluted following a 15-minute incubation at 65°C in Elution Buffer. Cross-links were reversed for both IP and Input samples by overnight incubation in a 65°C waterbath. On day 3, samples were treated with DNase-free RNase A (Thermo Fisher Scientific, EN0531) for an hour at 37°C, followed by Proteinase K (New England Biolabs, P8107S) for 2 hours at 37°C. Chromatin was purified with a Qiagen MinElute PCR Purification kit. qPCR reactions were performed with PowerUp SYBR Green Master Mix (Thermo Fisher Scientific, A25742) on an Applied Biosystems ViiA 7 Real-Time PCR system. ChIP-qPCR primers are listed in Supplemental Table 2C.

### Statistics.

Two-tailed, unpaired Student’s *t* tests were used for most analyses. One- and 2-way ANOVA with Tukey’s multiple-comparison tests were used when comparing more than 2 groups. *P* < 0.05 was used as the threshold for statistical significance. The exception to this is the RNA-Seq studies; statistics for the RNA-Seq analyses are detailed previously in Methods.

### Study approval.

All animal procedures and protocols in this study were approved by the Johns Hopkins IACUC and were performed in accordance with the *Guide for the Care and Use of Laboratory Animals* (National Academies Press, 2011).

### Data availability.

The RNA-Seq data discussed in this publication have been deposited in NCBI’s Gene Expression Omnibus (85) with the accession no. GSE236921. Analysis code is available on GitHub at https://github.com/hansenlab/fahrner_weaver (commit ID: 3868a2c). All other data associated with this study are present in the manuscript or supplemental materials; raw data are available in the Supporting Data Values file.

## Author contributions

JAF designed the CRISPR-Cas9 edit to generate the *Ezh2^R684C/+^* mouse and isolated MEFs. WYL and JAF performed the Western blot and initial mouse phenotyping. PK and RR collected and analyzed the μ-CT and dynamic bone histomorphometry data. WYL optimized conditions for osteoblast differentiation and prepared the libraries for untreated RNA-Seq. CWG performed GSK-J4 treatments, library preparation for drug-treated RNA-Seq, and ChIP-qPCR. CWG, LB, and KDH analyzed the RNA-Seq data. CWG wrote the manuscript. All authors contributed toward the editing and approval of this manuscript. HTB contributed essential resources at the start of the project. HTB and KDH participated in helpful discussions throughout. JAF conceived and directed the project and oversaw its completion.

## Supplementary Material

Supplemental data

Supplemental data set 1

Supporting data values

## Version 1 11/28/2023

In-Press Preview

## Version 2 01/09/2024

Electronic publication
